# Your baby has down syndrome: a reflexive thematic analysis of breaking the news to parents

**DOI:** 10.1186/s12884-025-07665-2

**Published:** 2025-05-06

**Authors:** Marcela Tenorio D, Johanna Sagner-Tapia, Renata Garibaldi, Vaso Totsika

**Affiliations:** 1 Centro de Investigación para la Mejora de los Aprendizajes, Facultad de Educación, Universidad del Desarrollo, Las Condes, Chile; 2 Millennium Institute for Care Research (MICARE), Santiago, Chile; 3 Núcleo de Ciencias Sociales y Humanidades, Universidad de la Frontera, Temuco, Chile; 4 Division of Psychiatry, University College London, London, UK

**Keywords:** Down syndrome diagnosis, Maternal experience, Healthcare communication, Prenatal diagnosis, Empathy, Support

## Abstract

The communication of a Down syndrome diagnosis to mothers is a pivotal moment that can impact their emotional well-being and the subsequent care for their child. This study aimed to explore maternal experiences with receiving a Down syndrome diagnosis. A total of 40 mothers participated, 42.5% received a prenatal diagnosis, 17.5% received the diagnosis during labor and delivery, and 40% received the information at the early postnatal period. Through reflexive thematic analysis, four key themes emerged: (1) About Who and How the News Was Delivered (2), About When the News Was Delivered (3), On Where the News Should Be Delivered (4), parents’ positive experiences emerging from this, predominantly, negative episode. Our findings emphasize the need for improved training for healthcare professionals in delivering sensitive diagnoses, with an emphasis on empathy, accurate information, and support resources for families.

**Supplementary Information:**

The online version contains supplementary material available at 10.1186/s12884-025-07665-2.

Informing a family that their unborn child has Down syndrome is a pivotal moment that can now occur not only at birth but also during the prenatal phase [1–4]. The way this news is delivered—how it is conveyed—holds significant weight for families and their decision to continue with the pregnancy [5–8].

The delivery of news in this context is crucial as it frames perspectives on disability, human nature, and parenthood [6, 9] and influences parental decisions, from pregnancy continuation to adjusting to the birth of a baby with Down syndrome [3, 10, 11].

In many countries, prenatal screening for Down syndrome is integrated into publicly funded healthcare programs and can be diagnosed with a degree of certainty [6, 10–12]. Studies indicate that while routine ultrasounds help parents see the fetus as a ‘child,’ deviations from typical development often cause disorientation or distress [1].

Approximately 85% of mothers of children with Down syndrome learn of the condition postnatally [13]. Prenatal diagnoses often occur through screening prompted by age-related risks or considerations about abortion [3, 10, 14, 15]. The communication of a Down syndrome diagnosis is shaped by stigma toward individuals with intellectual disabilities [16]. Intellectual disability, a developmental condition involving cognitive difficulties and challenges in adaptive behavior, is strongly associated with Down syndrome, the most common chromosomal genetic condition [17, 18]. Globally, people with intellectual disabilities are among the three most stigmatized groups, affecting their social inclusion [19, 20].

Some literature suggests that stigma toward intellectual disability may influence prenatal diagnosis processes, including in some cases implicit or explicit recommendations for abortion by physicians to mothers expecting a baby with Down syndrome for eugenic reasons [5, 21, 22]. Similarly, negative stigma may also affect postnatal diagnoses. Studies indicate that many physicians emphasize the limitations of intellectual disability when communicating the diagnosis [7, 15]. For some parents, the intellectual disability associated with Down syndrome is often the most devastating aspect of the news [23, 24].

Some authors stress that news-breaking should “help them [the parents] make the appropriate informed decision” (p.2) [6]. Therefore, the way the news is conveyed affects parental decisions about pregnancy continuation [5, 11, 21] and the postnatal bond, especially attachment [6, 8]. One study described mothers initially refusing to feed or care for the baby after learning of the Down syndrome diagnosis [7].

Delivering information clearly, calmly, with evidence-based details and space for parents to ask questions can foster a strong bond between healthcare professionals and parents, help manage the emotions triggered by unexpected news, and guide them through subsequent steps [7, 14, 15]. It is also relevant to recognise available evidence that shows that breaking such news is challenging for healthcare professionals [7].

There is limited evidence on how news of the birth of babies with Down syndrome is delivered globally. Most studies [14, 25–27], mainly conducted in Europe and North America, focus on elective abortion. These studies suggest that the delivery of the news often conveys a sense of bad news, reflecting negative attitudes and stigma toward disability [7, 15].

Evidence is limited for other countries. Chile has a high birth rate of babies with Down syndrome, at 2.4/1000 [28, 29], compared to the estimated 1/1000 prevalence rate in other countries [2], where rates are steadily decreasing. The country ranks third globally in Down syndrome births, after the United Arab Emirates and Japan [2]. In Chile, an abortion law was introduced in 2017, known as the “three causes law”. This law allows abortion when the pregnancy is the result of rape, or when the life of the fetus or the mother is at risk. Down syndrome is not considered a valid reason under these three cases, and therefore, abortion of fetuses with Down syndrome is prohibited and legally prosecuted. In addition to this, many healthcare professionals maintain conscientious objections, and negative perceptions of abortion persist [30]. Conscientious objection refers to the refusal to follow a norm due to moral or religious beliefs. Current legislation allows healthcare professionals to refuse abortions and to perform other procedures conflicting with their beliefs.

This study aimed to explore the experience of mothers receiving a Down syndrome diagnosis, either pre- or postnatally, contributing to the limited evidence in this field, including unique data from the Global South. A qualitative approach was used to capture these in-depth experiences.

## Methods

This qualitative study focused on the interactions between mothers and their children (aged 12–44 months) with Down Syndrome. The study employed a cross-sectional design and conducted a reflexive thematic analysis, framed within an interpretivist paradigm, to identify recurring themes in mothers’ recollections of the moment they were informed of their child’s diagnosis. In all cases included in the study, the mothers who received a prenatal diagnosis carried their pregnancies to term and have their babies alive and with them.

### Participants

This study was conducted with forty mothers (*n* = 40) of children with Down syndrome aged between 12 and 44 months old. All participants were living in an urban zone. The average age of the participating mothers was 37.7 years old (minimum 24 and maximum 45 years old), and of their babies was 20.4 months of age (minimum 12 and maximum 44 months old). Participants received prenatal and childbirth care in public hospitals (10%), private clinics (47.5%), a mix of public-private settings (22.5%), and 20% of participants did not specify the setting. Some details about the sociodemographic characteristics of the participants are presented in Table 1.

**Table 1:** Mothers’ sociodemographic data (*N* = 40)

		Percentage
**When the news was delivered**		
Prenatally	17	42.5%
Labour and Delivery	7	17.5%
Postnatally	16	40%
**Monthly family Income (USD)**		
< $181.15	1	2.5%
$181.16 < $357.16	1	2.5%
$357.17 < $566.87	7	17.5%
$566.88 < $909.87	2	5%
$909.88 < $2.439.954	10	25%
> $2478.44	19	47.5%
**Educational level**		
Incomplete elementary school	1	2.5%
Complete elementary school	0	0
Incomplete high school	0	0
Complete high school	8	20%
Technical degree	0	0
Graduate degree	21	52.5%
Postgraduate degree	10	25%
**Mothers’ pregnancy history**		
	Mean	
Age at the start of the pregnancy	37,7	
Gestational weeks	37,07	

### Procedure

The study was approved by the Accredited Scientific Ethics Committee. In Chile, these committees are accredited by the Ministry of Health. Participants were recruited through non-governmental organisations (NGOs) that provide therapeutic services to people with Down syndrome and their families. Field research experience in Chile has shown that this is the best strategy for recruiting participants with Down syndrome in scientific studies. Unlike other countries with higher income levels, in the Global South countries, most public policies are targeted rather than universal. In this case, not all children receive early stimulation, only those at risk for neurodevelopmental issues.

In practice, this means that any child with Down syndrome born in Chile can be referred by a pediatrician to the available care system called Chile Crece Contigo. However, there are waiting lists, and the quality of care has often been questioned, so most families complete their care by attending these NGOs. Within the NGOs, there is a wide range of costs; although they all offer more or less the same services for children under three years old, fees range from free care to payments around 650 dollars per month.

In coordination with the management of each NGO, families were provided with information about the study, and those who agreed to participate signed informed consent forms. Participants were also recruited through social media. In the sample reported in this study, six mothers were recruited through the Instagram community. In these cases, there were one-to-one meetings between the research team and the interested family, where the study objectives and scope were explained, and those who expressed willingness to participate signed informed consent forms.

From the grant formulation stage that enabled this study, an advisory group of five mothers of individuals with Down syndrome was established. Their sons and daughters ranged in age from 2 to 35 years at the time they were invited. Of these five mothers, four are professionals actively working, and one, the mother of the youngest child, is dedicated to his care. These mothers had previously participated in studies conducted by the research team. Their role was to accompany the researchers throughout the process, contributing insights from their own experiences. Following the Bigby et al. [31] model of participation for Experts by Experience, they served as advisors.

Once each participant agreed to be part of the study and signed the informed consent form, completed a questionnaire that collected socio-demographic data and this information was organized into a database. When the mothers answered the initial questionnaire, which included questions related to pregnancy and childbirth, it became evident that they had information about that time that they wanted to share. The researchers, together with the advisory group, decided to explore this in greater detail. To do so, they requested an amendment to the protocol submitted to the ethics committee to include questions aimed at obtaining this information. Once the committee’s authorization was obtained, three weeks after request itfollowing the initial request, this interview was added to the socio-demographic questionnaire, allowing the inclusion of all 40 participants.

These 40 mothers participated in a semi-structured interview aimed at exploring in greater depth their narratives about how they received the news that their children had Down syndrome. The interviews were conducted by a psychologist with specialist training in supporting families of children with intellectual disabilities. The semi-structured interviews began with an open-ended question that allowed participants to narrate their experiences freely. The interview then continued with a series of semi-structured questions to delve deeper into the mothers’ experiences. The interviews lasted a minimum of 15 min and a maximum of 40 min. The questionnaire used is available in Appendix 1. Each interview was transcribed by a research assistant, and the information was added to the database containing the socio-demographic data.

### Approach to analysis

Collected data were analysed using Reflexive Thematic Analysis [32]. This approach was considered appropriate for this study because it allows for both flexible and reflective work on a dataset containing information on a topic that has been scarcely addressed and that needs to be informed from epistemological perspectives that consider the territoriality of the Global South. As a flexible approach, it provides the possibility of developing a deductive analysis with a reflective positioning of the research team regarding the meanings conveyed in the interviews conducted.

The method was applied considering five of the six steps suggested by Braun & Clarke [32], which involved: a process of familiarization with the information contained in the database, initial coding, the generation of initial themes, the development and review of the themes, and finally, the refinement and labelling, which are presented here narratively.

All data were initially coded by one researcher and then discussed within the research team, including one researcher who had no prior involvement with the interviews. To enhance reflexivity and in an attempt to incorporate subjectivity, the team considered how their own experiences shaped interpretation. Additionally, we sought feedback from the external advisory group to strengthen interpretation through triangulation.

It is worth noting that the four authors of this article are women, three of whom are mothers who experienced their pregnancies in different parts of the world (Chile, the Netherlands, Germany and the United Kingdom). None of the authors is a mother of a child with Down syndrome. One of the authors is not a mother and approaches the process with greater distance. The participants were informed about the academic degrees and workplaces of the researchers, as well as the fact that the group included foreign and migrant researchers. However, they were not provided with information about the motherhood experiences of each researcher.

## Results

In all cases, the conversation began by asking the mothers where they had received the news regarding the presence of Down syndrome in their child. A total of 42.5% received a prenatal diagnosis, 17.5% received the diagnosis during labour and delivery, and 40% received the information at the early postnatal period.

**Fig. 1:**
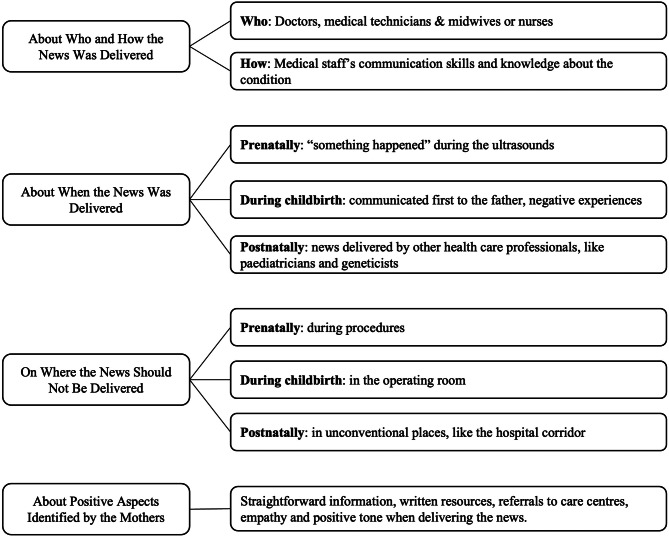
Conceptual map of the presented topics

The reflexive thematic analysis allowed the identification of four commonalities in the meanings conferred: [1] who and how the news was delivered (with two frequent subthemes being the skills and knowledge demonstrated at the time of communication) [2], when the news was delivered, with narratives appearing across three periods (prepartum, peripartum, and postpartum) [3], where news should not be delivered, with frequent references to the setting, and finally [4], positive experiences. A conceptual map of these topics is presented in Fig. 1.

### About who and how the news was delivered

Although who delivered the news and how was the process may seem like two separate topics, in the way the mothers provide information about it, these topics appear intertwined. It was not possible to separate them during the coding and analysis process; therefore, they are presented as interwoven as they appear in the narratives. We affirm that these topics are deeply intertwined because, in their narratives, the mothers describe who gave them the news and immediately include details or descriptors about how that moment unfolded.

Regarding who delivered the diagnosis, in 31 cases it was reported to be a doctor during a general consultation, in five cases an ultrasound technician, in two cases a nurse, and in two cases a person outside the immediate healthcare team.

A common theme that emerged in the interviews was that medical staff lacked both the skills and training to deliver this type of news, this included physicians, nurses and ultrasound technicians. Mothers pointed out that, on some occasions, the person who delivered the diagnosis provided incorrect information (such as: “*your child will never walk*” (or “*children with Down syndrome die very young*”). They even indicated at times that they could not provide more information due to a lack of knowledge about the condition and its prognosis. Several mothers mentioned that the news had been delivered in a manner they described as “*cold*,* with little empathy*.”

Regarding the skills of the doctors, one mother says:

*Doctors aren’t taught how to tell you that your baby has Down syndrome. They deliver the information as if it were a tragedy”* (35 years old, received care in a private clinic).

Regarding the skills of ultrasound technician and nurses, one mother says:

*The midwife spoke as if she were offering condolences”* (36 years old, did not specify where she received care).

Regarding knowledge, the mothers narrate that the healthcare staff seemed to be poorly informed about Down syndrome, making it difficult to provide accurate information. One mother said:

*The experience with the gynaecologist was different (compared to the neonatologist*,* who was ‘very empathetic and delicate in delivering information’) because he seemed not to be very informed about Down syndrome”* (43 years old, received care in a private clinic).

In another case, a mother recounted her experience with a geneticist consulted right after the birth:

*The geneticist at [Private Clinic] was a disaster*,* she was unpleasant. She said*,* ‘Well*,* at your age*,* you should have known something like this could happen*,*’ very unpleasant. Overall*,* it was catastrophic the entire time. She said*,* “Your child won’t eat*,* won’t breastfeed*,* and if he gets sick before his first year*,* he will die”* (43 years old, received care in a private clinic).

### About when the news was delivered

Several mothers mentioned that “*something happened*” during the ultrasound scan appointment, but that they did not receive clear information at that time. In the case of mothers who received the diagnosis prenatally, all of them recall having experienced situations that caught their attention. They mentioned things like: ‘*everyone in the room went silent and looked at each other*,’ ‘*the doctor appeared uncomfortable*,’ ‘*suddenly*,* they stopped talking to me*,’ ‘*they asked my companion to step out with the doctor for a moment*,’ among many others.

Some said, “*the doctor saw something*,* but didn’t want to tell me even when I asked*”, while others mentioned inappropriate comments from the doctor, such as “*he told me the baby was going to die*”. In several cases, mothers reported that during the ultrasound, they were warned about physical characteristics like *“he will be small”* or *“he will be short-legged*”, but no further information was provided.

Contrary, some mothers indicated being informed of signs suggestive of Down syndrome during a regular ultrasound check, but decided not to proceed with confirmatory tests. For example, one mother recounts:

*After the 16-week Doppler*,* they saw signs [suggestive of Down syndrome]*,* and I decided not to take the test [amniocentesis] and wait until birth”* (36 years old, received care both in a private clinic and public hospital).

A prenatal confirmation was not always well received:

*When we found out*,* we felt horrible. It was hard to accept the news. We decided not to seek information or help until the baby was born”* (35 years old, received care in public hospital).

Conversely, other mothers appreciated having learned about it prenatally, stating that it “*allowed them to arrive at the birth prepared and calm*” (39 years old, received care in a private clinic).

This diversity of experiences among mothers who found out their child’s condition prenatally contrasts with the narratives of mothers who received the news at the time of birth. For those mothers, it was a uniformly negative experience. Further, in 11 cases where the news was delivered at birth, it was first communicated to the father, who then informed the mother. Some narratives exemplify these experiences:

*They called the father after the birth to tell him that [the baby] had ‘traits of Down syndrome.’ They informed me after they told the father that [the baby] seemed to ‘have traits.’ They told me in the operating room while closing my abdomen*” (37 years old, received care in both in a private clinic and public hospital).

In another case, a mother recounts:

*While they were cleaning and weighing the baby*,* the paediatrician told the father*,* ‘Did you know your daughter has Down syndrome?’ Since it was during Covid times*,* the father had to tell me over the phone. The doctor was very cold when informing us*” (39 years old, did not specify where she received care).

Postnatal identification sometimes was done not by gynaecologists or midwives but by other health professionals such as paediatricians and geneticists. The experiences also varied widely. Some narratives described neutral experiences:

*The father found out first as the paediatrician told him. He was the one who informed me afterward”* (36 years old, did not specify where she received care).

However, other narratives are more striking. For example:

*During check-ups*,* they mentioned the risk of Down syndrome due to my age. However*,* ultrasounds and tests indicated everything was within parameters. When they took the baby after birth to clean her*,* and while I was waiting in the hospital corridor*,* a doctor approached and asked if I had been informed about Down syndrome. They then explained the suspicion and said they would conduct all tests. The baby was hospitalized in neonatology*,* so I was going in to give her milk. But they did not offer any further information. I remember a staff member*,* outside the hospital protocol*,* who was a mother of a person with Down syndrome*,* explained what was happening (…) it was difficult*,* I was very sad for over a year*” (42 years old, received care in a public hospital).

The fact that in 11 of the reported cases, the father received the initial news instead of the mother is one of the most striking findings of this study. Although no mother directly expressed emotions regarding this situation, there were indirect mentions that revealed mothers’ discomfort and surprise in response to it.

### On where the news should not be delivered

The narratives analysed highlighted wide variation in the location where the news is delivered. News is predominantly given during medical procedures for those who received it prenatally, in the operating room for those who received it during labour, and in unconventional places like hospital corridors for those who received it postnatally.

Regarding prenatal experiences, one mother shares:

*I found out when the technician told me during an ultrasound*,* before anything was confirmed*,* that the baby was going to die*” (35 years old, received care in a private clinic).

One mother recounts:

*They informed me …*,* saying that the girl ‘seemed to have traits*,*’ still in the operating room*,* while they were closing my abdomen. I would have liked them to wait a bit to tell me*,* a little more empathy from the doctors*” (37 years old, received care in both in a private clinic and public hospital).

In another case, a mother narrated the experience between her husband and a doctor:

*A doctor approached my husband in the hallway and asked if anyone had mentioned anything to him about Down syndrome. My husband didn’t know anything*,* and that’s when they explained the suspicion to him and said they were going to run all the tests”* (42 years old, received care in a public hospital).

### About positive aspects identified by the mothers

Although it was not common, in six interviews, the mothers highlighted certain aspects they found positive. While in all cases the experience of receiving the news was shocking and painful, it seems important to emphasize these moments, as they may provide insights into elements that should always be considered.

These narratives identified the following positive aspects: (a) that professionals provide clear and direct information about the child’s condition and its implications, (b) that professionals use expressions (both verbal and non-verbal) that convey calmness and de-escalate the situation, (c) that explicit information about care centers and support groups is provided, (d) that the family is informed about support and assistance opportunities for both the child and the immediate and extended family, (e) that the information is delivered in a positive tone, and (f) that, in general, the delivery of information is empathetic and cordial.

Here are two narratives that reflect these elements:

*When the doctor did the ultrasound*,* he was straightforward*,* told us what he saw*,* and that there was a probability of Down syndrome. He gave us information about the condition*,* mentioned various forms of support and therapies. It was a positive experience*” (38 years old, received care in a private clinic).

Another mother shares:

*When we received the news*,* a social worker came to talk to us*,* along with the doctor. The doctor gave us a book she had written about children with Down syndrome. She was very approachable*” (36 years old, received care both in a private clinic and public hospital).

## Discussion

The results of this study provide critical insights into the experience of mothers receiving a Down syndrome diagnosis for their child. These findings illustrate several important issues, particularly in how healthcare professionals communicate this diagnosis and the resulting emotional impact on families. The themes identified align with findings in previously published studies [23]. Available evidence suggests that these aspects have long-lasting implications for parents and families [33–36], and our results provide evidence in favor of the need and urgency for protocols in healthcare that ensure the delivery of information in a more humanized manner, addressing clear guidelines on who should deliver the information, where, and how.

One of the most notable findings concerns the intertwining of two key aspects: who delivered the diagnosis, and the communication skills demonstrated. Our results show that, although in most cases the person responsible for delivering the news was a medical doctor, there were cases where another professional delivered the news, which is noteworthy and suggests that there is neither clear regulation nor an established practice as a minimum standard. In all cases, this is with doctors, ultrasound technicians and nurses, the level of empathy, knowledge, and communication skills demonstrated were characterised by low levels of empathy and knowledge (about Down Syndrome) often resulting in negative experiences for participants.

This finding reflects similar evidence from other countries where healthcare professionals often lack adequate training in communicating sensitive information, particularly regarding genetic conditions like Down syndrome [13]. The high frequency of communicating incorrect or misleading information—such as dire predictions about the child’s future—highlights a crucial area for improvement. Healthcare professionals need to be equipped not only with accurate medical information but also with communication skills that reflect empathy and support for the family [37].

In this study, in 11 cases, the news was first delivered to the father, who then communicated it to the mother. This result is particularly striking and prompts several questions and reflections. Firstly, we cannot assert (or deny) that this is a widespread cultural practice, as we have not identified other studies providing comparative data. However, in a study involving the general population in the country, it was reported that fathers often have a private moment with their newborns, which is perceived as a highly positive experience for fostering bonding [38].

When mothers recount that the information was given to the father before them, they express dissatisfaction with this action, saying they felt “pasadas a llevar”—a colloquial expression in Chile used to refer to situations where essential rights have been disregarded. In this context, although it may seem striking, we suggest that this action constitutes a form of symbolic obstetric violence. Within the epistemological framework of obstetric violence, this practice is defined as the one exercised by healthcare personnel on women’s bodies and reproductive processes, characterized by dehumanizing treatment, excessive medicalization, and the pathologization of natural processes (for a complete revision with an idiosyncratic approach on Latin America review Beatriz vs. El Salvador, Inter-American Court of Human Rights [39]).

Obstetric violations may arise through omission or through deliberate action (e.g., actions performed without the woman’s consent, or mistreatment). Both mothers are fathers have an equal right to receive such news, and for this to take place in a compassionate and considerate manner.

Adhering strictly to the legal terms of the situation in Chile, in births, mothers are considered the patients, and the newborn is regarded as a subject of interest but not yet a rights-holder—a status that will be acquired upon registration, along with the right to identity. In this context, there is no doubt that the mother, as the patient, holds all rights, including the right to dignified, humanized, and well-informed care [40]. Practices where information is not provided, or incomplete information is given, constitute poor practices that, in this context, can be interpreted through feminist theories as forms of perpetuating social power relations that go beyond personal interactions. We believe this is a pivotal finding of our research that should prompt discussions on new guidelines and public policies aimed at the humanization of care in these situations.

The perception that some medical professionals approached the conversation as a “tragedy” underscores the detrimental effects of delivering the diagnosis in a way that can exacerbate distress for the family. The emotional tone set by healthcare providers is critical, as it may influence how parents process the diagnosis and begin to adjust their expectations for their child’s future [41]. Training programs for healthcare professionals should place a greater emphasis on empathy, sensitivity, and the provision of clear, accurate information, in addition to emphasizing the need for healthcare professionals to understand that there are factors related to how the news is delivered that can either complicate or protect the mental health of these mothers [42].

The timing of the diagnosis delivery appears to be another important factor influencing mothers’ experiences. While the literature has long debated the optimal moment for delivering difficult news [43], our findings suggest that mothers’ preferences varied but that communicating the diagnosis prenatally was, on average, better for mothers. Mothers who received a prenatal diagnosis had more time to prepare emotionally and practically, while those who were informed during birth often reported feelings of shock, grief, and unpreparedness. Mothers who were told prenatally expressed relief and appreciation for the time they had to process the information. These mothers reported feeling more “prepared and calm” at the time of their child’s birth, which contrasts with the overwhelming emotions experienced by mothers who received the news in the chaotic environment of the delivery room. There were still parents who found a prenatal diagnosis distressing, highlighting the need for healthcare providers to approach each family with sensitivity to their unique needs and circumstances, ensuring that both the timing and method of communication is appropriate [44].

In Chile, prenatal screenings do not have the primary objective of detecting chromosomal abnormalities in the fetus to allow mothers to consider an elective abortion for this reason. This contrasts significantly with several European countries, where clear guidelines exist for the prenatal diagnoses of conditions such as Down syndrome, giving parents sufficient time to make decisions, including abortion. In some cases, the option of abortion is even encouraged once the diagnosis is confirmed [10, 45]. Even in those cases where the prenatal diagnosis was confirmed or suspected, maternal narratives do not reveal reflections on the possibility of terminating the pregnancy. Instead, the timing of the diagnosis in our study is mostly about how mothers and families prepare emotionally and practically for the baby’s arrival, whether through information seeking or by postponing decisions until after birth.

The setting where the diagnosis was delivered also played a critical role in shaping mothers’ experiences. Many of the mothers who received the diagnosis during labor or delivery or in a hospital corridor described these locations as inappropriate for such a significant moment. The chaotic nature of these environments, combined with the physical and emotional strain of childbirth, contributed to negative experiences. This finding supports existing recommendations that difficult news should be delivered in a private, calm, and controlled environment where parents can process the information without additional stressors [46].

Despite the overwhelmingly negative narratives, a small number of mothers described positive experiences in receiving the diagnosis, indicating that good practices do exist. These experiences were often characterized by clear, accurate information, delivered in a compassionate manner, while mothers were also provided with resources for future support. This finding highlights the need to not only focus on the content of the communication but also on the manner in which healthcare professionals deliver the news. This finding is consistent with studies that emphasize the importance of compassionate communication in healthcare settings, particularly in situations involving genetic conditions [47].

Our results show that there is a clear need for training of healthcare staff that are met with the task of informing families that their baby has Down syndrome. There are existing protocols that provide valuable guidance on how to convey bad or fatal news, covering aspects such as the appropriate manner, setting, and timing for communication, as well as the rights of the patient (e.g. PEWTER [48]; SHARE [49]; and GRIEV_ING [50]).

Whilst these protocols were not created with Down syndrome in mind, there are some important aspects that could be taken from them, for example PEWTER [48], contains a step called PREPARE, which expects medical staff to be aware of their own biases, beliefs, and values to adequately convey the news. We suggest that it is important to consider that these protocols were not designed to deliver the news of the birth of a child with a neurodevelopmental condition. Therefore, future research should aim to provide evidence to establish the feasibility of using these methods.

Findings of positive experiences provide valuable insight into what parents view as best practice when receiving a Down syndrome diagnosis. Findings highlight the need for professionalism, empathy, and the provision of clear and actionable information about the condition and available resources.

While the results presented contribute to an area that has scarcely been explored in Latin America, with a large sample considering it is a qualitative approach, the study does have limitations. Firstly, the design was restricted in scope as the qualitative study was designed in direct response to emerging findings from the ongoing quantitative study with support from the parent advisory team. While it was a timely opportunity to investigate parents’ experiences of receiving the diagnosis, future studies specifically focused on exploring this topic should take a more theoretically guided approach, for example, considering more psycho-social variables. Secondly, the sample was not intentionally balanced in variables that might be important, such as obstetric service type (public versus private) and family socio-economic level. In our study, 50% of the mothers came from low- and middle-income backgrounds, and 50% from high-income backgrounds. In Chile, approximately 60% of the population has low or middle income. In Latin America, those with higher incomes tend to access better healthcare services [51], and inequalities in health service access are large. Therefore, if the challenges described are present in this sample, it is reasonable to assume that families with lower incomes will be experiencing similar, if not greater, challenges. A prospective longitudinal investigation, following mothers from when they receive the diagnosis until their child reaches the first year of life, might provide more insight into the impact of the first communication.

## Conclusion

Findings from the present study highlight the, predominantly, distressing nature of receiving a diagnosis of Down syndrome for parents in Chile. The variability in mothers’ experiences underscores the need for standardized, empathetic communication protocols in healthcare settings. Healthcare professionals must be equipped with the necessary skills and knowledge to deliver such news compassionately and accurately. Training programs should focus on increasing staff knowledge about Down syndrome and, in parallel, on the emotional nuances of delivering a diagnosis, ensuring that all families receive the support they need during this critical moment.

As this is a qualitative analysis, we did not explore the role of specific demographic characteristics in the theme generation, though at the same time this was not a finding that emerged either. It would be interesting for future, quantitative studies to consider the role of context more systematically. Future research should explore how healthcare providers can better tailor their communication approaches to individual families’ needs, as well as how interventions might be designed to improve communication practices in settings where such diagnoses are commonly delivered. By addressing these issues, we can move toward a healthcare system that offers parents not only accurate information but also the emotional support necessary to navigate this life-changing event.

## Electronic supplementary material

Below is the link to the electronic supplementary material.

Supplementary Material 1

## Data Availability

The data collected in this study are not publicly accessible, to protect the confidentiality of the participants. However, the corresponding author may be contacted and upon reasonable request the data set can be made available.

## References

[1] Åhman A, Edvardsson K, Fagerli TA, Darj E, Holmlund S, Small R, et al. A much valued tool that also brings ethical dilemmas - a qualitative study of Norwegian midwives’ experiences and views on the role of obstetric ultrasound. BMC Pregnancy Childbirth. 2019;19(1):33. 30651083 10.1186/s12884-019-2178-x PMC6335783

[2] Al-Biltagi M. Down syndrome from epidemiologic point of view. EC Pediatr. 2015;1(2):82–91.

[3] Crombag NMTH, Boeije H, Iedema-Kuiper R, Schielen PCJI, Visser GHA, Bensing JM. Reasons for accepting or declining down syndrome screening in Dutch prospective mothers within the context of National policy and healthcare system characteristics: a qualitative study. 2016. 10.1186/s12884-016-0910-3 PMC4880977 27229318

[4] Matamala P, Barrera C, Valdes E, Pedraza D, Manieu D, Parra M. P030: implementation of nuchal translucency screening in the detection of major congenital structural defects and chromosomal abnormalities in a Chilean population. Ultrasound Obstet Gynecol. 2003;22(S1):79–79.12858309

[5] Badke HE, Cooley K, Luce CE. Down syndrome and the importance of a neutral diagnosis delivery: A community service project. Bethel University; 2022.

[6] Gueneuc A, Dagher C, Rameh G, Haddad G, Hivernaud D, Mousty E, et al. Announcing fetal pathology: challenges encountered by physicians and potential role of simulation in training for breaking bad news. J Gynecol Obstet Hum Reprod. 2021;5(4):1–5. 10.1016/j.jogoh.2020.102044 33346160

[7] Jain R, Thomasma DC, Ragas R. Down syndrome: still a social stigma. Am J Perinatol. 2002;19(2):99–107. 11938484 10.1055/s-2002-23553

[8] Mugweni E, Goodliffe S, Jaswal S, Walker M, Emrys-Jones A, Adams C, et al. Improving the way healthcare professionals deliver different news to families during pregnancy or at birth: a qualitative study. Prim Health Care Res Dev. 2021;22:e10. 33775272 10.1017/S1463423620000651 PMC8101073

[9] Shakespeare T. Disability: the basics. Routledge; 2018.

[10] Rosman S. Down syndrome screening information in midwifery practices in the Netherlands: strategies to integrate biomedical information. Health: Interdisciplinary J Social Study Health Illn Med. 2016;20(2):94–109. 10.1177/1363459314561695 25504473

[11] Thorneycroft R, Prenatal, Testing. Down syndrome, and selective termination: A (Critical) criminology of genocide?? In: Silva DMD, Deflem M, editors. Diversity in criminology and criminal justice studies. Emerald Publishing; 2022. pp. 167–81.

[12] Ngan OMY, Yi H, Bryant L, Sahota DS, Chan OYM, Ahmed S. Parental expectations of Raising a child with disability in decision-making for prenatal testing and termination of pregnancy: A mixed methods study. Patient Educ Couns. 2020;103(11):2373–83. 32507714 10.1016/j.pec.2020.05.010

[13] Skotko BG, Capone GT, Kishnani PS. Postnatal diagnosis of down syndrome: synthesis of the evidence on how best to deliver the news. Pediatrics. 2009;124(4):e751–8. 19786436 10.1542/peds.2009-0480

[14] Skotko BG. Prenatally diagnosed down syndrome: mothers who continued their pregnancies evaluate their health care providers. Am J Obstet Gynecol. 2005;192(3):670–7. 15746657 10.1016/j.ajog.2004.11.001

[15] Skotko BG, Kishnani PS, Capone GT. Prenatal diagnosis of down syndrome: how best to deliver the news. Am J Med Genet A. 2009;149A(11):2361–7. 19787699 10.1002/ajmg.a.33082

[16] Piepmeier A, Estreich G, Adams R, Unexpected. parenting, prenatal testing, and down syndrome. NYU; 2021.

[17] Schalock RL, Luckasson R, Tassé MJ. AAIDD manual: Intellectual disability: definition, diagnosis, classification, and systems of supports. 12th Edition. AAIDD; 2021. 10.1352/1944-7558-126.6.439 34700345

[18] Antonarakis SE, Skotko BG, Rafii MS, Strydom A, Pape SE, Bianchi DW et al. Down syndrome. Nature reviews disease primers 2020 6:1 [Internet]. 2020 Feb 6 [cited 2024 Oct 10];6(1):1–20. Available from: https://www.nature.com/articles/s41572-019-0143-7 10.1038/s41572-019-0143-7 PMC8428796 32029743

[19] Pelleboer-Gunnink HA, van Weeghel J, Embregts PJCM. Public stigmatisation of people with intellectual disabilities: a mixed-method population survey into stereotypes and their relationship with familiarity and discrimination. Disabil Rehabil [Internet]. 2021 [cited 2021 Jun 24];43(4):489–97. Available from: https://www.tandfonline.com/action/journalInformation?journalCode=idre20 10.1080/09638288.2019.1630678 31242402

[20] Scior K. Toward Understanding Intellectual disability stigma: Introduction. In: Scior K, Werner S, editors. Intellectual disability and stigma [Internet]. London: Palgrave Macmillan, London; 2016 [cited 2023 Aug 30]. pp. 3–13. Available from: https://link.springer.com/chapter/10.1057/978-1-137-52499-7_1

[21] Asgarova S. Mothers’ experiences of continuing their pregnancy after prenatally receiving a diagnosis of down syndrome. University of British Columbia; 2019.

[22] Vuk M. Reconsidering disability, friendship and Otherness– Theological and ethical perspectivesv. University of Fribourg; 2019.

[23] Grane FM, Lynn F, Balfe J, Molloy E, Marsh L. Down syndrome: parental experiences of a postnatal diagnosis. J Intellect Disabil. 2023;27(4):1032–44. 35698902 10.1177/17446295221106151 PMC10647884

[24] Jain PD, Nayak A, Karnad SD, Doctor KN. Gross motor dysfunction and balance impairments in children and adolescents with down syndrome: a systematic review. Clin Exp Pediatr. 2022;65(3):142–9. 34126707 10.3345/cep.2021.00479 PMC8898616

[25] de Graaf G, Buckley F, Skotko BG. Estimation of the number of people with down syndrome in Europe. European journal of human genetics [Internet]. 2021 Mar 1 [cited 2024 Aug 20];29(3):402. Available from: https://doi.org//pmc/articles/PMC7940428/10.1038/s41431-020-00748-y PMC7940428 33130823

[26] Grossman TB, Chasen ST. Abortion for Fetal Genetic Abnormalities: Type of Abnormality and Gestational Age at Diagnosis. Am J Perinatol Rep [Internet]. 2020 [cited 2024 Aug 20];10:87–92. 10.1055/s-0040-1705173 10.1055/s-0040-1705173 PMC7075712 32190411

[27] Caban-Holt A, Head E, Schmitt F. Down syndrome. In: Rosenberg R, Pascual J, editors. Rosenberg’s molecular and genetic basis of neurological and psychiatric disease. 5th ed. London, UK: Academic Press Inc.; 2014. pp. 163–70.

[28] Nazer J, Cifuentes L. Prevalencia al nacimiento de malformaciones congénitas en las maternidades chilenas participantes en el ECLAMC en el período 2001–2010. Rev Med Chil [Internet]. 2014 [cited 2023 Aug 23];142(9):1150–6. Available from: http://www.scielo.cl/scielo.php?script=sci_arttext%26pid=S0034-98872014000900009%26lng=es%26nrm=iso%26tlng=pt 10.4067/S0034-98872014000900009 25517055

[29] Rivera M, Solari G, Peralta M, Rivera M, Solari G, Peralta M. Estudio de prevalencia en niños recién nacidos con síndrome Down y sus características antropométricas. Hospital Regional de Antofagasta, Chile. Revista chilena de nutrición [Internet]. 2021 [cited 2024 Mar 19];48(2):238–44. Available from: http://www.scielo.cl/scielo.php?script=sci_arttext%26pid=S0717-75182021000200238%26lng=es%26nrm=iso%26tlng=es

[30] Pérez B, Sagner-Tapia J, Elgueta HE. Despenalización Del aborto En Chile: Una Aproximación Mixta desde La percepción Del aborto En Población comunitaria. Gac Sanit. 2020;34(5):485–92. 30583975 10.1016/j.gaceta.2018.11.004

[31] Bigby C, Frawley P, Ramcharan P. Conceptualizing inclusive research with people with intellectual disability. J Appl Res Intellect Disabil [Internet]. 2014 Jan [cited 2025 Jan 20];27(1):3–12. Available from: https://pubmed.ncbi.nlm.nih.gov/24390972/10.1111/jar.12083 24390972

[32] Braun V, Clarke V. Conceptual and design thinking for thematic analysis. Qualitative Psychol. 2022;9(1):3–26.

[33] Clark L, Canary HE, McDougle K, Perkins R, Tadesse R, Holton AE. Family sense-making after a down syndrome diagnosis. Qual Health Res [Internet]. 2020 Oct 1 [cited 2024 Oct 10];30(12):1783. Available from: https://doi.org//pmc/articles/PMC7814853/10.1177/1049732320935836 PMC7814853 32618226

[34] Cunha AMFV, Blascovi-Assis SM, Fiamenghi GA. [Impact of delivering the news about Down syndrome on parents: life stories]. Cien Saude Colet [Internet]. 2010 [cited 2024 Oct 10];15(2):445–51. Available from: https://pubmed.ncbi.nlm.nih.gov/20414612/10.1590/S1413-81232010000200021 20414612

[35] Muggli EE, Collins VR, Marraffa C. Going down a different road: first support and information needs of families with a baby with Down syndrome. Medical Journal of Australia [Internet]. 2009 Jan 1 [cited 2024 Oct 10];190(2):58–61. Available from: https://onlinelibrary.wiley.com/doi/full/10.5694/j.1326-5377.2009.tb02275.x 10.5694/j.1326-5377.2009.tb02275.x 19236288

[36] Sheets KM, Baty BJ, Vázquez JC, Carey JC, Hobson WL. Breaking difficult news in a cross-cultural setting: A qualitative study about Latina mothers of children with Down syndrome. J Genet Couns [Internet]. 2012 Aug 7 [cited 2024 Oct 10];21(4):582–90. Available from: https://link.springer.com/article/10.1007/s10897-011-9425-2 10.1007/s10897-011-9425-2 22147086

[37] Sharkiya SH. Quality communication can improve patient-centred health outcomes among older patients: a rapid review. BMC Health Serv Res. 2023;23(1):886. 37608376 10.1186/s12913-023-09869-8 PMC10464255

[38] Herrera F. A horror movie with a happy ending’: childbirth from the father’s perspective. NORMA. 2020;15(3–4):251–66.

[39] Corte Interamericana de Derechos Humanos. Caso Beatriz Y Otros VS. El Salvador. 2023.

[40] Chadwick R. Ambiguous subjects: obstetric violence, assemblage and South African birth narratives. Fem Psychol. 2017;27(4):489–509.

[41] Lerwick JL. Minimizing pediatric healthcare-induced anxiety and trauma. World J Clin Pediatr. 2016;5(2):143. 27170924 10.5409/wjcp.v5.i2.143 PMC4857227

[42] Kammes RR, Lachmar EM, Douglas SN, Schultheiss H. Life-altering: A qualitative analysis of social media birth stories from mothers of children with down syndrome. J Intellect Disabil. 2022;26(4):919–37. 35898186 10.1177/17446295211025960

[43] Kitz CC, Barclay LJ, Breitsohl H. The delivery of bad news: an integrative review and path forward. Hum Resource Manage Rev. 2023;33(3):100971.

[44] Roodbeen R, Vreke A, Boland G, Rademakers J, van den Muijsenbergh M, Noordman J, et al. Communication and shared decision-making with patients with limited health literacy; helpful strategies, barriers and suggestions for improvement reported by hospital-based palliative care providers. PLoS ONE. 2020;15(6):e0234926. 32559237 10.1371/journal.pone.0234926 PMC7304585

[45] Thompson E, Brett J, Burns E. What if something goes wrong? A grounded theory study of parents’ decision-making processes around mode of breech birth at term gestation. Midwifery [Internet]. 2019 Nov 1 [cited 2024 Oct 20];78:114–22. Available from: https://pubmed.ncbi.nlm.nih.gov/31421541/10.1016/j.midw.2019.08.006 31421541

[46] Seifart C, Falch M, Wege M, Maier RF, Pedrosa Carrasco AJ. NEO-SPEAK: A conceptual framework that underpins breaking bad news in neonatology. Front Pediatr. 2022;10:1044210. 36440326 10.3389/fped.2022.1044210 PMC9681898

[47] Jeffrey D. Empathy, sympathy and compassion in healthcare: Is there a problem? Is there a difference? Does it matter? https://doi.org/101177/0141076816680120 [Internet]. 2016 Dec 6 [cited 2024 Oct 20];109(12):446–52. Available from: https://journals.sagepub.com/doi/full/10.1177/0141076816680120 10.1177/0141076816680120 PMC5154411 27923897

[48] Nardi T, Keefe-Cooperman K. Communicating bad news: A model for emergency mental health helpers. Int J Emerg Mental Health Hum Resil. 2006;8(3):203–7. 16944793

[49] Fujimori M, Shirai Y, Asai M, Kubota K, Katsumata N, Uchitomi Maiko Fujimori Y, et al. Effect of communication skills training program for oncologists based on patient preferences for communication when receiving bad news: A randomized controlled trial. J Clin Oncol [Internet]. 2014;32:2166–72. Available from: www.jco.org. 24912901 10.1200/JCO.2013.51.2756

[50] Hobgood C, Harward D, Newton K, Davis W. The educational intervention ‘GRIEV_ING’ improves the death notification skills of residents. Acad Emerg Med. 2005;12(4):275–301. 15805319 10.1197/j.aem.2004.12.008

[51] Houghton N, Bascolo E, Del Riego A. Socioeconomic inequalities in access barriers to seeking health services in four Latin American countries. Revista Panamericana de Salud Pública [Internet]. 2020 May 8 [cited 2025 Apr 9];44:e11. Available from: www.10.26633/RPSP.2020.11 10.26633/RPSP.2020.11 PMC7055457 32165889

