# Supplementary material for: Your baby has down syndrome: a reflexive thematic analysis of breaking the news to parents
Source: BMC Pregnancy Childbirth. 2025 May 6;25:536. doi: 10.1186/s12884-025-07665-2 (PMC12054234; doi:10.1186/s12884-025-07665-2)
Supplement: Supplementary file 1 — Supplementary Material 1 [file 12884_2025_7665_MOESM1_ESM.docx]

**Question Guide**

**Baby’s Information:**

- Name:
- Age (months):
- Date of birth:

**Mother’s Information:**

- Name:
- Age:
- Region of residence:
- Educational level:
  - Incomplete primary
  - Completed primary
  - Incomplete secondary
  - Completed secondary
  - Technical
  - Undergraduate
  - Postgraduate

**Total household income (CLP):**

- Less than $178,334
- Between $178,335 and $351,613
- Between $351,614 and $558,069
- Between $558,070 and $895,744
- Between $895,745 and $2,439,954
- Greater than $2,439,955

**Pregnancy Background:**

- Age at the start of pregnancy:
- Age at delivery:
- Weeks of gestation:
- Type of delivery (vaginal/cesarean):
- Were there complications during delivery? Yes/No
- What kind?

**Down Syndrome Identification:**
During your pregnancy and delivery, did you receive care in:

- Public healthcare
- Private healthcare
- Mixed system

Was there prenatal identification?

- Yes
- No

When were you informed?

Who informed you?

**Ask mothers if they have comments about the experience of receiving the identification:**

- Can you tell me about your experience of receiving the news?

Additionally, you can ask these questions if they don’t arise naturally during the mother’s account:

- How do you think the healthcare staff handled delivering the information to you?
- Compared to your other pregnancies, did you notice any differences in the ultrasounds or in the way the doctors behaved? (ask if they have other children).
